# Reply to: Letter to the Editor: More is More? Total Pancreatectomy for Periampullary Cancer as an Alternative in Patients with High-Risk Pancreatic Anastomosis: A Propensity Score-Matched Analysis, by Marchegiani, Giovanni et al.

**DOI:** 10.1245/s10434-022-11468-6

**Published:** 2022-02-25

**Authors:** Sebastian Hempel, Florian Oehme, Jürgen Weitz, Marius Distler

**Affiliations:** Department of Visceral, Thoracic and Vascular Surgery, University Hospital and Faculty of Medicine Carl Gustav Carus, Technische Universität Dresden, Dresden, Germany

We thank the authors for their interest in our recent study on total pancreatectomy for periampullary cancer as an alternative treatment option for patients with high-risk pancreatic anastomosis.^1^ We greatly appreciate the opportunity to respond to their comments.

In our study, we analyzed the oncologic and postoperative outcome of pancreaticoduodenectomy (PD) and total pancreatectomy (TP). We found no differences in the median overall and progression-free survival or in the administration of adjuvant therapy. Moreover, postoperative morbidity and mortality were comparable in the two groups. We concluded that primary TP may provide a safe treatment alternative to pancreatic head resection, especially for selected patients with high-risk pancreatic anastomosis or preoperatively impaired glucose tolerance.

As highlighted in our discussion section, one major limitation of our study was the heterogeneity between the patient cohorts regarding tumor localization and extension of resection, which was reflected in the concomitant arterial reconstructions in the TP group, for example. Naturally, the indications and surgical complexity of TP represent a very wide and variable field, so differentiation of various subgroups of TP, as described by Loos et al.,^2^ is useful for better risk stratification. Regardless, in our study, complex TP, even with arterial vascular replacement, was not inferior to pancreatic head resections in terms of postoperative outcomes.

We agree with the authors that consideration of the risk stratification for postoperative pancreatic fistula (POPF) would make the analysis even more valuable. Unfortunately, due to some missing fistula risk score (FRS) data in the pylorus-preserving pancreaticoduodenectomy/Whipple control group, especially in older cases, it was not feasible to include one of the fistula risk scores as a propensity score-matching variable. However, considering all the available FRS data, both groups did not differ significantly with regard to POPF risk, displaying an intermediate risk in all three scores (FRS, alternative fistula risk score, and updated alternative fistula risk score).

It is undisputed that TP cannot be considered as an alternative for all patients undergoing pancreatic head resection due to the lifelong consequences of pancreatic insufficiency. Recently, three studies compared this particular group of high-risk PD procedures with TP in terms of surgical outcome, and reported mainly better results after TP.^3–5^ Regarding quality of life, a retrospective analysis by Marchegiani et al.^5^ described comparable results after TP and high-risk PD. To prevent pancreoprivic diabetes or to preserve at least partial endogenous insulin secretion to mitigate the psychosocial aspects of diabetes or lifelong insulin therapy, intraportal islet autotransplantation should be also considered.^6^ Therefore, we are eagerly awaiting the results of the PAN-IT trial (NCT01346098) and further studies on TP with islet autotransplantation (IAT) that also take into account oncologic issues.

In conclusion, as the authors have already emphasized, the most crucial factor lies with the careful patient selection. In this context, the recently published results of the International Study Group of Pancreatic Surgery (ISGPS)^7,8^ could presumably contribute to an even better selection of those patients for whom TP, if applicable with IAT, should be considered instead of high-risk PD.
